# Supplementary material for: The dependency structure of the financial multiplex network model: New evidence from the cross-correlation of idiosyncratic returns, volatility, and trading volume
Source: PLoS One. 2025 Apr 18;20(4):e0320799. doi: 10.1371/journal.pone.0320799 (PMC12007721; doi:10.1371/journal.pone.0320799)
Supplement: S1 File — The Supporting Information, S1 File, contains additional tables and graphs for the robustness analysis. (PDF) [file pone.0320799.s001.pdf]

Supporting information for: The dependency structure of the financial multiplex network model: New evidence from the cross-correlation of idiosyncratic returns, volatility, and trading volume

Appendix A

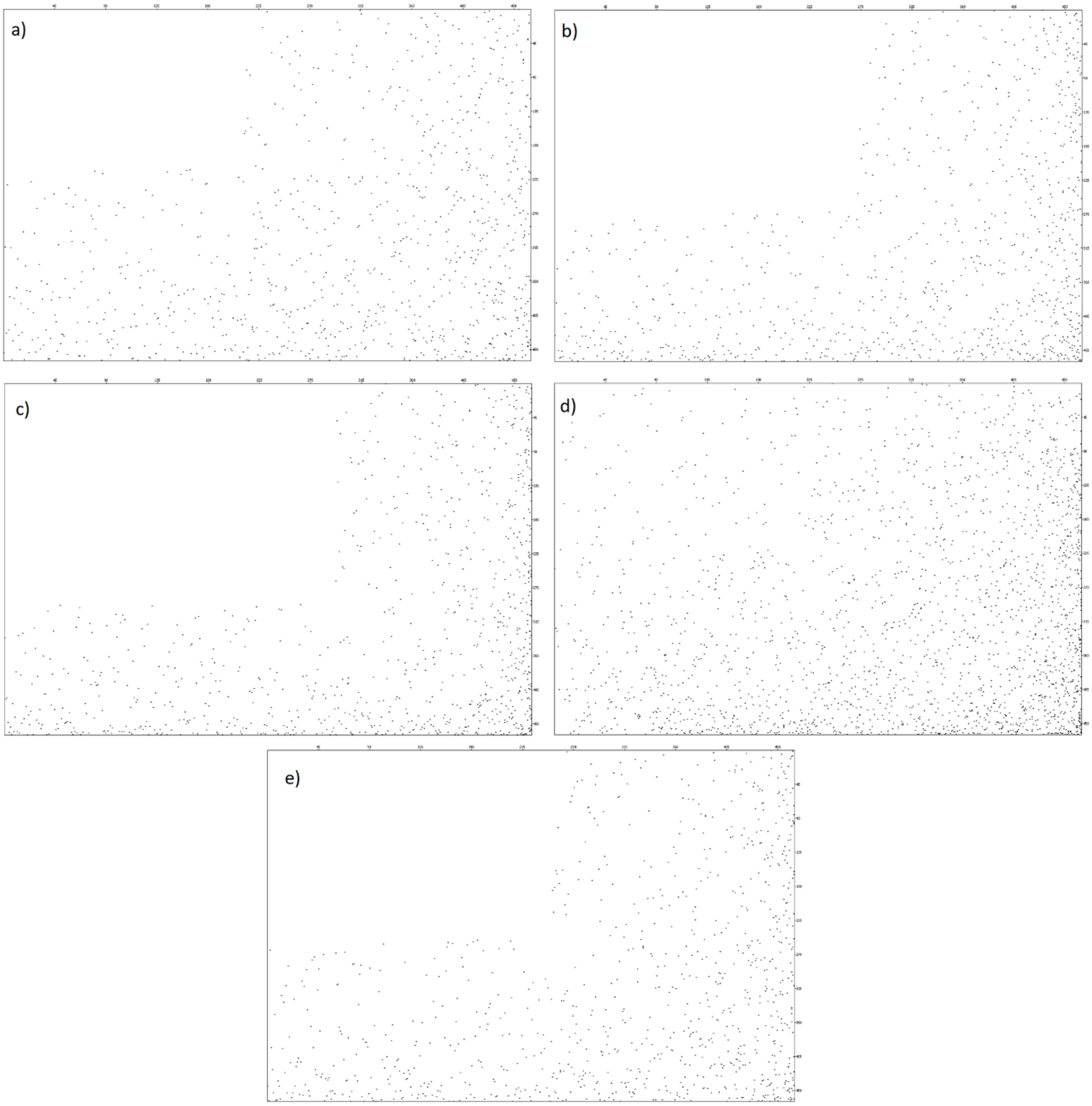

Fig A1. Heat map of the network of (a) idiosyncratic return; (b) volatility; (c) trading volume; (d) multiplex (FMN); (e) cross-correlation of stock return (SRN)

Appendix B

Table B1. Examination of power-law properties of network quantities

Network	N	m	Lower bound x_{min}	Exponent of the power-law γ	p-value	Kolmogorov-Smirnov statistic	Fraction of nodes with degree $(k \leq 8)$
**Multiplex**	465	2 112	4	3.029	0.714	0.025	91.6%
**Idiosyncratic return**	465	464	6	4.921	0.691	0.038	99.6%
**Volatility**	465	464	2	2.534	0.836	0.032	97.6%
**Trading volume**	465	464	3	2.781	0.117	0.062	97.8%
**Stock return**	465	464	3	3.023	0.520	0.043	98.7%

Number of iterations for each network – 930,000; N – number of vertices; m – number of edges

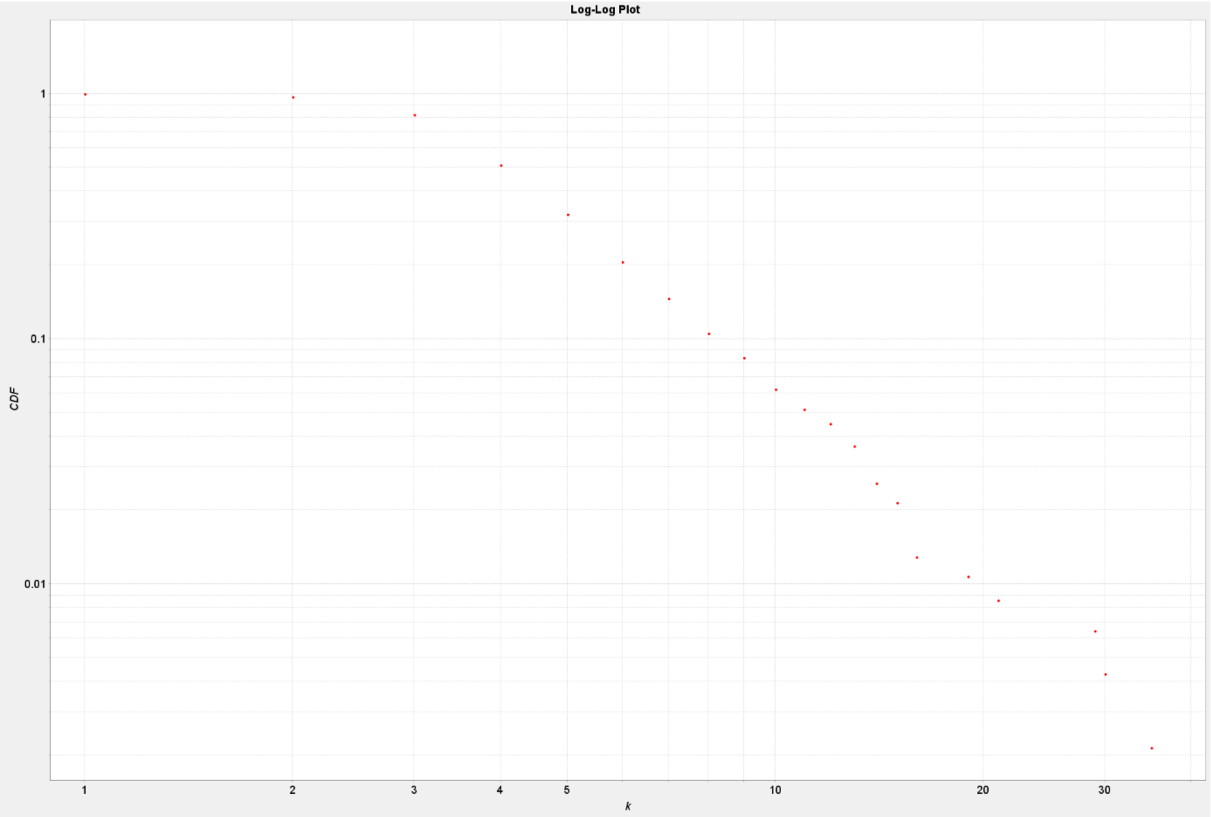

Fig B1. The degree distribution of the financial multiplex network (FMN)

CDF – complementary cumulative distribution function

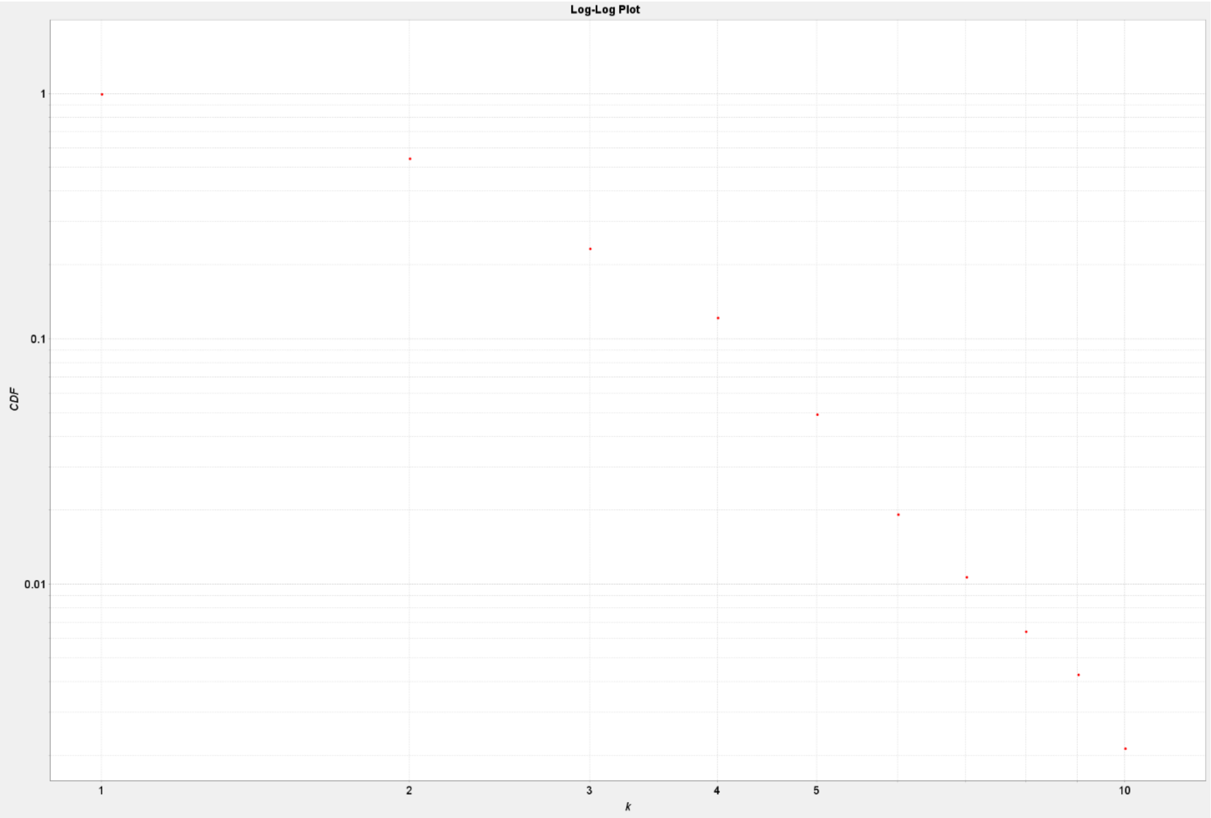

Fig B2. The degree distribution of the idiosyncratic return network (layer 1)
CDF – complementary cumulative distribution function

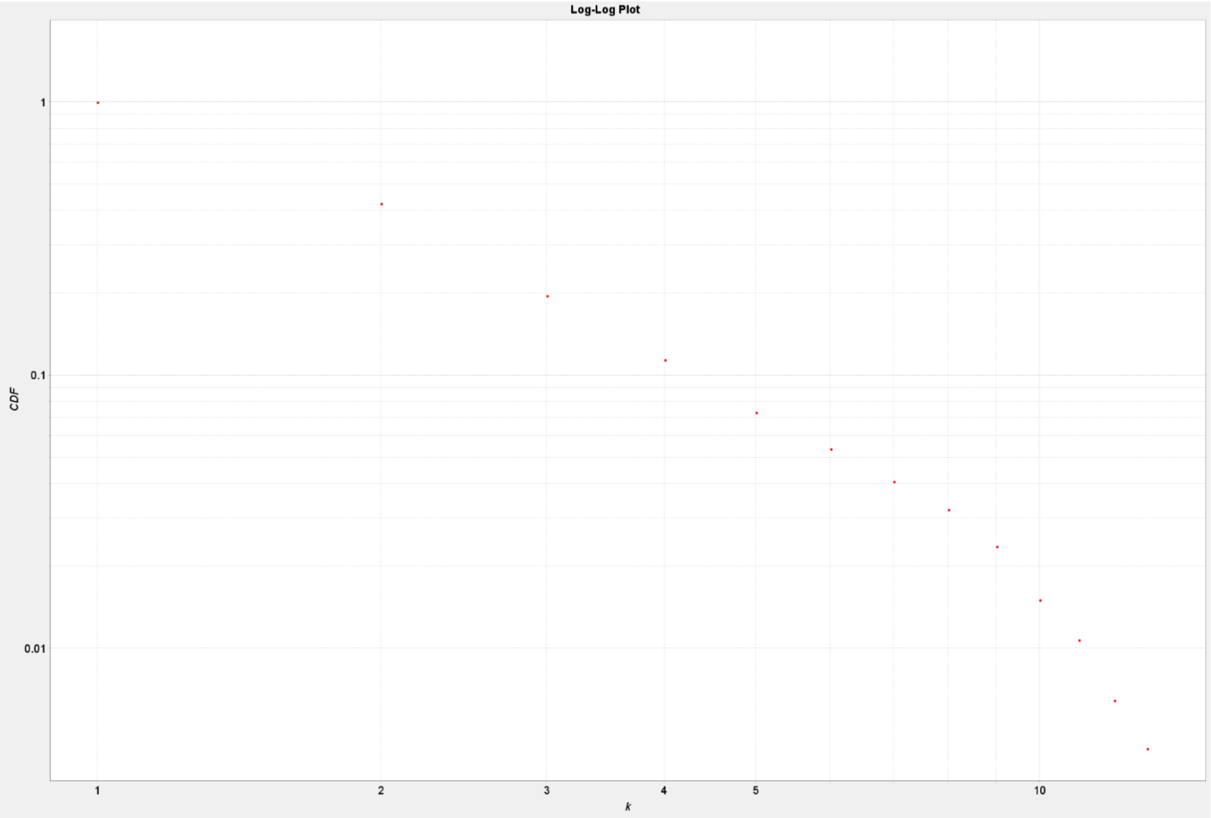

Fig B3. The degree distribution of the volatility network (layer 2)
CDF – complementary cumulative distribution function

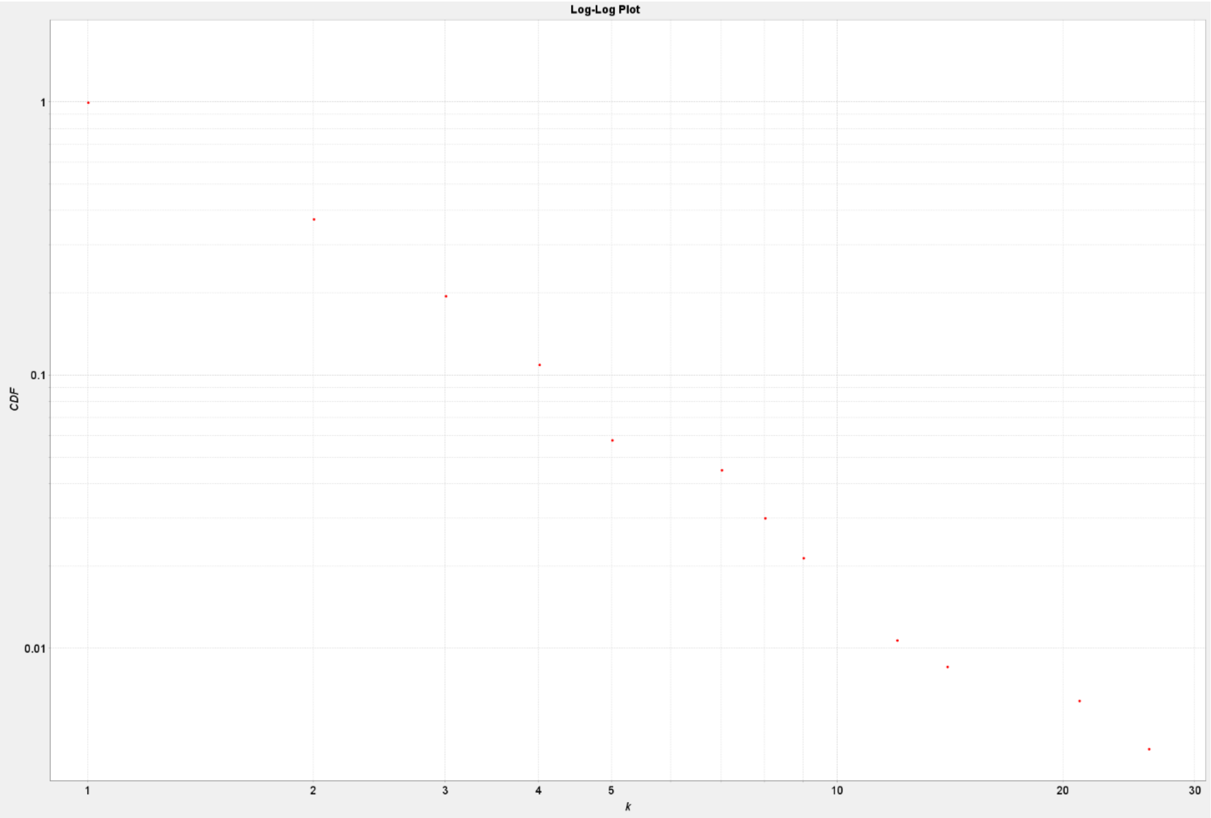

Fig B4. The degree distribution of the trading volume network (layer 3)
CDF – complementary cumulative distribution function

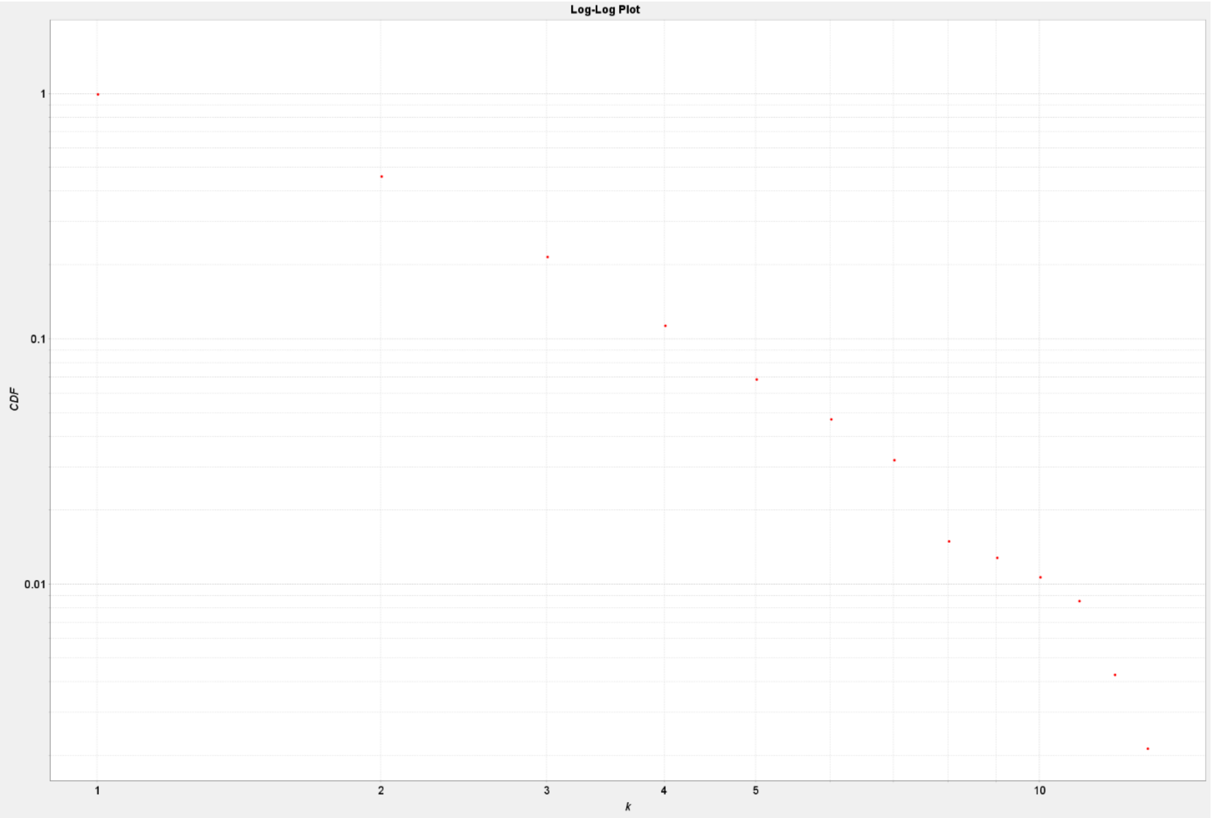

Fig B5. The degree distribution of the stock return network (SRN)
CDF – complementary cumulative distribution function

Appendix C

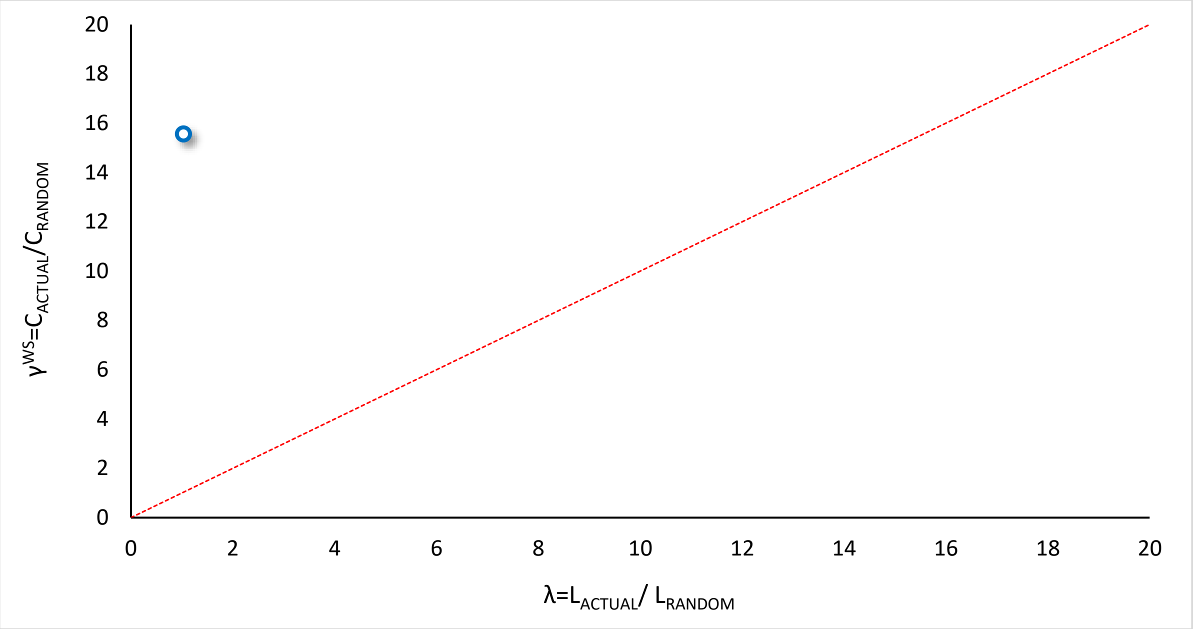

Fig C1. The indicator of the S-W network in the sense of Watts–Strogatz

Appendix D

Table D1. Edge overlapping ratio of the layers of the FMN and the SRN

Network	Idiosyncratic return	Volatility	Trading volume	Stock return
**Idiosyncratic return**	-			
**Volatility**	0.429	-		
**Trading volume**	0.233	0.244	-	
**Stock return**	0.700	0.466	0.228	-
