# Supplementary material for: The dependency structure of the financial multiplex network model: New evidence from the cross-correlation of idiosyncratic returns, volatility, and trading volume
Source: PLoS One. 2025 Apr 18;20(4):e0320799. doi: 10.1371/journal.pone.0320799 (PMC12007721; doi:10.1371/journal.pone.0320799)
Supplement: Attachment — Submitted filename: Referee_ENG.pdf [file pone.0320799.s002.pdf]

Report on:
The dependency structure of the financial multiplex network model

December 10, 2024

Summary

The manuscript proposes to understand equity markets using a multiplex with an original three-layer structure: minimal spanning tree (MST) of returns purged of the systematic component, MST of volatilities, and MST of trading volumes.
The author proposes a comparative investigation of the network properties of the various layers of the multiplex, of the multiplex summarized by the recurrence of edges between pairs of layers (Financial Multilateral Network, FMN), and of the classical MST of time series of returns (stock return network, SRN). In particular, he finds the properties of i) small world and ii) robustness to node deletion, in the case of the FMN exclusively. He also finds, again in the case of FMN, a better out-of-sample predictability, and a better ability to predict the Sharpe ratio.
All these elements are a priori interesting. However, an overall investigation of their robustness remains to be carried out, in particular to the structure of the database and its pre-processing, the choice of graph models, and the ability of the FMN to represent equity markets with a relevant sectoral structure. A systematic review in relation to benchmark portfolio theories could usefully structure this revision work.
Furthermore, the interpretation of certain results is questionable. In particular, the small world and node deletion robustness properties of FMN do not prove that this model represents the underlying structure of equity markets in a more relevant way than other reference graphs, in the absence of consensus on the usual verification of these properties within financial networks.

Main remarks

1. A main shortcoming of the manuscript is that the contribution is not situated in relation to theoretical portfolio models, which are nonetheless its background. The literature in the field of complex systems is extensively described, but that of finance is barely mentioned.

2. In connection with the previous point, the author states that complex systems constitute a well-established research paradigm for studying financial data structures containing large amounts of financial information, which is debatable. It is probably more accurate to see complex systems as a modeling method adapted, in particular, to the multi-dimensionality and non-linear dynamics of financial systems. This clarification would enable the reader to situate the manuscript's methodological contribution more appropriately.

3. As a prerequisite to the investigation of graph indicators, an assessment of the relevance of the FMN is missing. For example, by verifying the sectorial assortativity property usually sought in econo-physics. This assessment should at least be carried out by visual inspection, coloring nodes according to sectors (see minor remark 1).

4. The study period is long and marked by major macroeconomic and financial shocks (2013-2019 for set 1, and 2018-2023 for set 2). To support the static graph approach, it would be necessary to prove that there are no significant breaks in the structure of the dynamic graph over the period (e.g. by analyzing time series of topological indicators).

5. The fact that the synthetic representation of the multiplex possesses the small-world property is not an argument in favor of the proposed method: there is no evidence that the underlying data structure does

indeed possess this property. Furthermore, the "small world" property can be caused by the cyclicity of a graph (investigated here) but also by its desassortativity (not investigated as contributing to the "small world structure"). Finally, it is impossible to characterize a financial network with the "small-world" property as "efficient" (p. 22). Firstly, because in finance theory, the notion of efficiency refers to a market where prices reflect available information, which has nothing to do with it. Secondly, because theoretical models of financial crises show that, in extreme episodes, it is on the contrary the modularity of networks that best enables crises to be absorbed.

6. The binarization of MST layers and the recurrence graph that summarizes the multiplex is not discussed. However, it is neither justified by the readability of the graphs nor by any computational gain. Furthermore, are the results robust to this choice?

7. The differences in statistical properties found between classical MST, FMN layers and FMN itself are generally a direct consequence of the properties of the MST, in particular its acyclicity. The author himself notes this on several occasions. FMN, on the other hand, has a purely endogenous structure. To appreciate this structure as a whole, it would be useful to provide a readable representation (e.g. using a force-directed layout). However, this method only works if the graph has not been binarized first.

8. The result of multiplex network regression analysis (Table 6) is largely determined by the way the problem is framed: even in out-of-sample, the FMN is likely to be better explained than the SRN, since the exogenous variables are the layers of the FMN. Similarly, the Sharpe ratio (Table 8) is necessarily better explained by the FMN indicators, which include volatility and the risk-free rate (which participate in its calculation), than by the SRN indicators, which only include returns.

9. The study of robustness to network failures is not convincingly justified. If the graph model is relevant and this robustness is found, then the market is resilient to failures (node deletions). But the robustness to failures result alone does not prove the superiority of the model with which it is obtained, contrary to what is claimed.

Minor remarks

1. Figures are difficult to read (non-vector format, low resolution, no sector identification by node color, no asset tickers even for hubs).

2. The definition of the content of the mutiplex layers comes late (third page of the introduction).

3. It is understandable that the returns layer should be built from returns purged of the common component, to avoid too strong a correlation with the volatility layer. However, the approach of searching for the *common component* of the *specific factors* of returns is partly counter-intuitive and deserves to be discussed. Furthermore, the low similarity of the volatility and volume layers raises questions about indicators of both variables. It is likely that the similarity of the distribution tails would be much stronger. This observation brings us back to the absence of any prior study of the non-linearities of the data used. The proposed literature review also mentions various works focusing on distribution tails and extreme risks.

4. The notion of noise (in particular p. 4) to characterize graph density is confusing. In the field of dynamic graphs (and signal processing), noise characterizes the volatility of measured data (edges of the computed graph) compared with the true structure of the data (edges of the underlying graph).

5. The literature review is largely presented as a succession of summaries. It would be more informative to shorten it by grouping the works into major families of methods.

6. Different stages of data processing should be discussed. In particular, i) the OLS estimator used for the returns layer is almost certainly biased on the long financial series employed (p. 12). To get an idea of this, it would be useful to provide a time-series representation of the residuals; and ii) the choice of volatility measure is not justified.

7. The acronyms used in the tables (definitions are given below) do not make for optimal readability.

8. In Tables 4 and 5, is it correct that the similarity measures (correlations and Jaccard index) of the layers with the FMN are all identical, whereas the bilateral similarities between layers are different? It would also be useful to calculate the Jaccard of the three layers without the FMN (and not just the three layers and the FMN).

9. The introduction provides some answers to the research questions mentioned in the introduction: it would be useful to reiterate these questions.
