# Supplementary material for: The dependency structure of the financial multiplex network model: New evidence from the cross-correlation of idiosyncratic returns, volatility, and trading volume
Source: PLoS One. 2025 Apr 18;20(4):e0320799. doi: 10.1371/journal.pone.0320799 (PMC12007721; doi:10.1371/journal.pone.0320799)
Supplement: Attachment — Submitted filename: Response to Reviewers.docx [file pone.0320799.s003.docx]

January 16, 2025

**Point-by-point response to Reviewers**

**Editor comment:** In your revision you need to address fully all the comments from both reviewers. I particularly advise you to try to make your paper more targeted and emphasize what is its novelty. You may consider adjusting the title to reflect that. Furthermore, I suggest to add a more elaborated conceptualization and literature reviews suggested by one of reviewers and more critical approach to your methodology, as well as more appropriate interpretation of results. Finally, additional robustness tests suggested by one of the reviewers, would greatly enhance the contribution of your manuscript.

**Responses to Editor**

Dear Editor:

Thank you very much for the referee report on my manuscript “The dependency structure of the financial multiplex network model”. The article has been revised in accordance with the recommendations set forth by the reviewers.

The revised manuscript includes:

1) restructuring the literature review and conducting an additional literature review (related to portfolio optimization),
2) corrections, extensions, and additional explanations and justifications of the adopted research methodology,
3) revisions to the interpretation of the results obtained,
4) additional robustness tests.

Furthermore, in response to your advice, the title of the manuscript has been expanded to: “The dependency structure of the financial multiplex network model: New evidence from the cross-correlation of idiosyncratic returns, volatility, and trading volume”.

Please find enclosed a detailed, point-to-point response to all Reviewer comments with all changes clearly specified. Changes in the text are marked with colors, as follows: Reviewer #1: red; Reviewer #2: blue.

I wish to take this opportunity to thank the Reviewers for their time, effort and insightful comments that have resulted in an improved manuscript. I would also like to thank you for handling my paper. Hopefully, my revised manuscript can be regarded as meeting the high standards of PlosOne journal.

With kind regards

Corresponding author

**Point-to-point responses to the Reviewers**

**Response to Reviewer #1:**

I would like to thank the referee because he/she provided a considerable number of comments to improve my paper. The newly added and significantly revised portions in the paper are marked in red to make them easy to identify.

**Comment 1:** *“explain better in which sense (and provide much more focused literature holding the idea that) financial markets are "naturally" multiplex.”*

**Reply:** The explanation referring to the natural representation of financial markets as a multiplex network has been included.

**page 10, line 218**

“The stock market can naturally be represented as a multiplex network.”

**is revised into,**

“The stock market can naturally be represented as a multiplex network due to the complex and multi-layered interactions among various financial instruments, institutions, and agents. The natural multiplexity of financial markets arises from the coexistence of diverse and interrelated layers of interaction. This framework allows for the integration of heterogeneous and simultaneously relationships between market participants, capturing their complex interdependencies and the multidimensionality of financial interactions. In the context of financial markets, each layer of the multiplex network can include different types of dependencies [54], highlighting the multifaceted nature of interactions among entities [55]. The interbank market is another example of a naturally multiplexed network representation [13], or, in a broader context, a model of a multilayer financial network that considers different types of interactions among banks, capital markets, and other market participants [56]. Multiplex networks consist of layers that coexist, interact, and evolve within a broader complex system, each characterized by distinct structures and functions [57]. Another study [58] analyzes financial networks as multilayer structures, examining various types of dependencies among financial institutions. The work [23] proposes a framework for a multiplex network, which is based on stock dependencies, sector-specific and location-specific layers.”

**Comment 2:** *“I think that in this paper a section devoted to the consequent application to simplicial complexes cannot be missing. At the end, although the manuscript is a nice piece to read, it still lacks "the innovative" strike. In spite of the fact that the author tries to underline the innovative nature of the work, a true innovation would be in the field of simplicial complexes, which are attracting growing attention in network theory.”*

**Reply:** Thank you for this important suggestion. I agree that the field of simplicial complexes offers significant application possibilities in the context of analyzing network structures. However, given the context of the research, the aim of the study, the chosen research methodology, and in particular the lack of an explicit application of the simplicial complexes approach, it seems that embedding the innovation of the conducted study in the field of simplicial complexes might not be the most appropriate approach. Nevertheless, the Reviewer's remark is highly relevant, and its incorporation has been reflected in the context of directions for future research.

**page 43, lines 967-969**

“Future studies should aim to more deeply analyze the relationships between the financial multiplex network and firm performance, such as stock returns, and the volatility of the stock price. A network dynamic approach could be applied in this area.”

**are revised into,**

“Future studies should aim to more deeply analyze the relationships between the financial multiplex network and firm performance, such as stock returns, and the volatility of the stock price. A network dynamic approach could be applied in this area. One promising direction for future research involves exploring the application of simplicial complexes to evolving multilayer networks, particularly in contexts where temporal and higher-order interactions play a crucial role. Simplicial complexes provide a means of capturing both local and global properties of networks, thereby facilitating a more profound analysis of these systems [111]. These mathematical constructs are instrumental in modeling and analyzing complex structures within networks. Moreover, simplicial complexes extend the analytical capabilities of financial multilayer networks, enabling the simultaneous modeling of multilateral interactions both within individual layers and between them. They also facilitate the representation of interactions among multiple entities, capturing relationships that extend beyond pairwise connections. This capability is particularly advantageous in complex systems where higher-order interactions play a significant role [112,113].”

**Response to Reviewer #2:**

Thank you for your valuable feedback on my manuscript. Your insightful comments and suggestions have been invaluable in enhancing the quality of our work.  Text changes are marked in blue.

**Major remarks:**

**Comment 1:** *“A main shortcoming of the manuscript is that the contribution is not situated in relation to theoretical portfolio models, which are nonetheless its background. The literature in the field of complex systems is extensively described, but that of finance is barely mentioned.”*

**Reply:** The introduction of the manuscript has been revised to include a discussion of the study's positioning within the existing portfolio optimization literature. This addition situates the study within the broader academic context by emphasizing a network approach in portfolio optimization.

**page 3**

**An additional paragraph has been added:**

“Network models play a crucial role in portfolio optimization by providing insights into the structure and dynamics of asset relationships within complex financial systems. Network models allow for a detailed mapping of how asset returns are interconnected and illustrate the dependency between network topology and its function, particularly in the context of financial networks. The structure of a network can significantly influence risk evaluation and asset performance in portfolio construction [26–29]. Financial network models significantly enhance portfolio optimization by providing a framework to analyze the complex interrelationships between assets [30], facilitating the identification of high-performing investments [31], and improving the robustness of portfolio strategies through dynamic analysis and noise reduction. Network peripherality serves as an important indicator for identifying optimal assets [32,33]. The recent studies reveal that peripheral nodes in a network can potentially yield better portfolio performance compared to central nodes [11,34–36], where the risk-return ratio plays a pivotal role. Stocks located in the network's peripheral region exhibit a more diversified composition and demonstrate reduced vulnerability to irregular stock price fluctuations during market volatility [8]. This insight allows for the selection of assets that are more profitable, well-diversified, and less risky. On the other hand, the superiority of portfolios based on peripheral stocks or centrality vertices is contingent upon the prevailing conditions of the stock market over a specified time horizon [37,38]. This indicates that this area has not been well explored. Nonetheless, empirical results of the study [39] demonstrated that network-based approaches yield better out-of-sample performance compared to traditional pairwise correlation methods.”

**Comment 2:** *“In connection with the previous point, the author states that complex systems constitute a well-established research paradigm for studying financial data structures containing large amounts of financial information, which is debatable. It is probably more accurate to see complex systems as a modeling method adapted, in particular, to the multi-dimensionality and non-linear dynamics of financial systems. This clarification would enable the reader to situate the manuscript's methodological contribution more appropriately.”*

**Reply:** The introduction about complex networks has been rephrased according to the Reviewer’s suggestions.

**page 1, lines 20-21; 29-31**

“Complex networks are a well-established research paradigm for studying financial data structures containing large amounts of information. The complex network approach is a powerful analytical framework that has been used to study and understand a wide range of complex systems [1,2].”

**are revised into,**

“~~Complex networks are a well-established research paradigm for studying financial data structures containing large amounts of information.~~ Complex networks have emerged as a robust modeling methodology, particularly adept at capturing the multidimensionality and nonlinear dynamics inherent in financial systems. The intricate interconnections and interdependencies within financial markets necessitate analytical frameworks capable of representing such complexity. Recent studies have demonstrated the effectiveness of complex network approaches in modeling the multifaceted nature of financial markets, providing insights into systemic risk and market behavior [1]. Additionally, the nonlinear interactions prevalent in financial systems have been effectively analyzed using complex network methodologies, offering a deeper understanding of market dynamics [2,3]. The complex network approach is a powerful analytical framework that has been used to study and understand a wide range of complex systems [4,5].”

**Comment 3:** *“As a prerequisite to the investigation of graph indicators, an assessment of the relevance of the FMN is missing. For example, by verifying the sectorial assortativity property usually sought in econo-physics. This assessment should at least be carried out by visual inspection, coloring nodes according to sectors (see minor remark 1).”*

**Reply:** The preliminary analysis of the network, including the FMN, the SRN, and the three FMN layers, has been extended to verify the sectoral assortativity property. The results confirm the observed tendency in the study to connect vertices in the financial network based on sectoral classification. The analysis was performed computationally to preserve precision, and as a complementary step, by visually inspecting and coloring the nodes by sector. For more details on modifying network graphs, see the response to minor remark #1.

**page 27, after line 617**

“A preliminary analysis of the networks confirms the presence of sectoral assortativity (see Table 3). A positive assortativity coefficient indicates that networks exhibit assortative behavior, characterized by a tendency to form connections between assets belonging to the same sector. This property is most prominent in the idiosyncratic return layer. Previous research has demonstrated that stocks in correlation-based networks consistently form clusters closely aligned with economic sector classifications [6,28,46,75,92,94,101–105]. Likewise, the results are statistically significant when employing the QAP approach.

Table 3. Results of sector assortativity mixing

| **Network** | **Observed r** | **Expected r** | **Std. Dev.** | **P (expected r** $\boldsymbol{\geq}$ **Observed r)** | **P (expected r = Observed r)** | **P (expected r** $\boldsymbol{\leq}$ **Observed r)** |
| --- | --- | --- | --- | --- | --- | --- |
| **Multiplex** | **0.539** | -0.003 | 0.030 | 0.000 | 0.000 | 1.000 |
| **Idiosyncratic return** | **0.811** | -0.003 | 0.046 | 0.000 | 0.000 | 1.000 |
| **Volatility** | **0.618** | -0.004 | 0.045 | 0.000 | 0.000 | 1.000 |
| **Trading volume** | **0.425** | -0.006 | 0.046 | 0.000 | 0.000 | 1.000 |
| **Stock return** | **0.755** | -0.004 | 0.046 | 0.000 | 0.000 | 1.000 |

Number of QAP iterations = 930,000

**Comment 4:** *„The study period is long and marked by major macroeconomic and financial shocks (2013-2019 for set 1, and 2018-2023 for set 2). To support the static graph approach, it would be necessary to prove that there are no significant breaks in the structure of the dynamic graph over the period (e.g. by analyzing time series of topological indicators).”*

**Reply:** I agree with the reviewer's comment that the study period (set 1 over set 2) overlaps with periods of market disruption, such as the Chinese stock market crisis (2015-2016), the global public health crisis due to the COVID-19 pandemic in 2020, and geopolitical tensions related to the Russian-Ukrainian armed conflict in 2022. In my estimation, it is not feasible to demonstrate the absence of significant disruptions to the structure of the dynamic graph during the period under consideration. In other words, this would contradict the results of many studies on the impact of the aforementioned events on the functioning of financial markets and, consequently, on the financial network's structure during these extreme periods. However, it should be pointed out that the adopted sufficiently long period (set 2) for the constructed networks, including a sufficiently long auxiliary period for the calculation of idiosyncratic stock returns (set 1), significantly “dilutes” potential structural breaks in the network structure resulting from short-term shocks. Furthermore, a retrospective analysis reveals that such periods of market disruption occur intermittently. Consequently, they should be regarded as an inherent feature of the functioning of financial markets over an extended timeframe. For instance, it would be feasible to extend the study's time period. However, this would necessitate the consideration of additional market stresses, such as the sovereign debt crisis (2010-2012) or the market collapse associated with the Global Financial Crisis (2008-2010). Considerations aside, this is a rather interesting thought. The reviewer's comment has been pointed out as a limitation of the study.

**page 43, line 950**

“This research has several limitations. (…).”

**is revised into,**

“This research has several limitations. (…). Furthermore, such extreme events in financial markets have the potential to trigger significant structural breaks in networks. However, the adopted long study period should adequately mitigate their impact.”

**Comment 5:** *“The fact that the synthetic representation of the multiplex possesses the small-world property is not an argument in favor of the proposed method: there is no evidence that the underlying data structure does indeed possess this property. Furthermore, the "small world" property can be caused by the cyclicity of a graph (investigated here) but also by its desassortativity (not investigated as contributing to the "small world structure"). Finally, it is impossible to characterize a financial network with the "small-world" property as "efficient" (p. 22). Firstly, because in finance theory, the notion of efficiency refers to a market where prices reflect available information, which has nothing to do with it. Secondly, because theoretical models of financial crises show that, in extreme episodes, it is on the contrary the modularity of networks that best enables crises to be absorbed.”*

**Reply:** The study of the small-world property of the multiplex network was not intended to demonstrate the superiority of this network form over the SRN. In this context, the goal was to highlight the differences in the architecture of these networks, complementing previous analyses that focused on the demonstrated differences in the statistical properties of the networks.

**page 40, lines 894-897**

“In contrast, the multiplex network has none of the typical structures, has a weaker community structure with fewer modules, and has less pronounced disassortative behavior. Additionally, only the multiplex network displays the small-world property. These observations indicate that different networks can exhibit unique structural and behavioral characteristics and have distinct properties, highlighting the importance of understanding the underlying phenomena they represent.”

**are revised into,**

“In contrast, the multiplex network has none of the typical structures, has a weaker community structure with fewer modules, and has less pronounced disassortative behavior. Additionally, only the multiplex network displays the small-world property, which results from the nature of its construction. This does not imply that the MLN outperforms the SRN. These observations indicate that different networks can exhibit unique structural and behavioral characteristics and have distinct properties, highlighting the importance of understanding the underlying phenomena they represent.”

I admit that the term "efficient" was used misleadingly, although in the context of the network. The sentence has been corrected.

**page 29, line 651-652**

“Short cuts play a crucial role in creating a more efficient and interconnected network.”

**are revised into,**

“Short cuts play a crucial role in creating a more ~~efficient~~ compact and interconnected network.”

**Comment 6:** *“The binarization of MST layers and the recurrence graph that summarizes the multiplex is not discussed. However, it is neither justified by the readability of the graphs nor by any computational gain. Furthermore, are the results robust to this choice?”*

**Reply:** The rationale for using the binarization approach to construct MST-based networks has been added. Furthermore, an additional section (4.6. Robustness analysis) has been added to demonstrate the robustness of the adopted network dichotomization solution.

**page 15, lines 351-353**

“The adjacency matrix $\boldsymbol{A}=\left\{ a_{ij} \right\}$ of a monoplex network is the $N$-dimensional, unweighted, and symmetric matrix with elements

$a_{ij}\boldsymbol{=}\left\{ \begin{aligned} 1 if there is an edge between stocks i \mathrm{and}j \left( i\neq j \right) \\ 0 othewise \end{aligned} \right.\left( i, j=1,2,\ldots,N \right)$	(1)

**are revised into,**

“The adjacency matrix $\boldsymbol{A}=\left\{ a_{ij} \right\}$ of a monoplex network is the $N$-dimensional, unweighted, and symmetric matrix with elements

$a_{ij}\boldsymbol{=}\left\{ \begin{aligned} 1 if there is an edge between stocks i \mathrm{and}j \left( i\neq j \right) \\ 0 othewise \end{aligned} \right.\left( i, j=1,2,\ldots,N \right)$	(1)

The adoption of a binary representation of financial networks is justified by several advantages over weighted networks that retain the distance metric on the edges. First, unweighted networks derived from the distance matrix simplify the complexity of the network's structure by reducing the granularity of edge weights. This approach focuses solely on the presence or absence of relationships between assets rather than their precise strength or magnitude. Furthermore, unweighted networks are inherently robust to minor fluctuations and noise in the underlying distance metrics. In financial datasets, the inherent noise and vulnerability to minor fluctuations in correlations can lead to potential overinterpretation of trivial variations in edge weights. By employing binarization, this risk is significantly reduced, thereby enhancing the reliability of the resulting network which effectively delineates only the most critical structural characteristics of the system, thus laying a more dependable foundation for subsequent analysis. Finally, unweighted networks present a uniform framework for comparative analyses across various layers of multiplex network. Weighted networks, by their very nature, complicate such comparisons because of the discrepancies in edge weights that do not have a direct comparability in different contexts. In other words, the added dimension of weighted networks brings with it more complexity, which may hide the basic topology and not allow direct comparison.”

**page 37**

**An additional section “4.6. Robustness analysis” has been added**

**“4.6. Robustness analysis**

The fundamental analysis was conducted through the simplification of a network approach, which entailed the dichotomization of the MSTs obtained using the distance metric $d_{ij}$ and the subsequent construction of an unweighted multiplex network. The robustness test is applied to the relaxation of the network binarization assumption, resulting in the formation of a multiplex network, its corresponding individual layers, and the SRN as a weighted network. It should be stressed that comparisons of the statistical properties of the networks were omitted, as weighted and unweighted networks inherently differ. The robustness test is applied to the analyses presented in Sections 4.3, 4.4, and 4.5.

Table 10 shows the results of the correlations between the weighted networks. The results do not reveal significant differences compared to the basic analyses. It should be pointed out that the assessment of network similarity, as measured by Jaccard similarity, Czekanowski-Sørensen-Dice matching coefficients, and EOR, yields identical values. This is because the evaluation considers only the presence of edges between nodes in the network.

Table 10. Pearson correlation coefficients (weighted networks)

| **Network** | **Multiplex** | **Idiosyncratic return** | **Volatility** | **Trading volume** |
| --- | --- | --- | --- | --- |
| **Multiplex** |  |  |  |  |
| **Idiosyncratic return** | 0.749* |  |  |  |
| **Volatility** | 0.676* | 0.355* |  |  |
| **Trading volume** | 0.683* | 0.181* | 0.201* |  |
| **Stock Return** | 0.575* | 0.632* | 0.398* | 0.181* |

* denotes significant level of 0.1% based on the QAP with 9,300 iterations, where the probability that the observed correlation coefficient is greater than expected is 1.000.

The results obtained for network regression (Table 11) and the threshold for robustness to failure (Table 12) in weighted networks are similar to those obtained for unweighted networks. This indicates the robustness of the results with respect to the assumption of dichotomization of the networks at the final stage of their construction.

Table 11. Network regression results (weighted networks)

| MODEL 1 | Dependent variable: Multiplex network | Coefficient | P (expected >= observed) | P (expected = observed) | P (expected <= observed) |
| --- | --- | --- | --- | --- | --- |
|  | Constant | 0.001*** (0.000) | - | - | - |
|  | Idiosyncratic return | 0.716*** (0.003) | 0.000 | 0.000 | 1.000 |
|  | Volatility | 0.591*** (0.003) | 0.000 | 0.000 | 1.000 |
|  | Trading volume | 0.781*** (0.003) | 0.000 | 0.000 | 1.000 |
|  | *F-test* | 520,726.109*** | 0.000 | 0.000 | 1.000 |
|  | *R^2^* | 0.879 |  |  |  |
| MODEL 2 | **Dependent variable: Stock return network** | **Coefficient** | **P (expected >= observed)** | **P (expected = observed)** | **P (expected <= observed)** |
|  | Constant | 0.001*** (0.000) | - | - | - |
|  | Idiosyncratic return | 0.432 *** (0.002) | 0.000 | 0.000 | 1.000 |
|  | Volatility | 0.199*** (0.003) | 0.000 | 0.000 | 1.000 |
|  | Trading volume | 0.032*** (0.002) | 0.000 | 0.000 | 1.000 |
|  | *F-test* | 55,638.772*** | 0.000 | 0.000 | 1.000 |
|  | *R^2^* | 0.436 |  |  |  |
| MODEL 3 | **Dependent variable: Multiplex network** | **Coefficient** | **P (expected >= observed)** | **P (expected = observed)** | **P (expected <= observed)** |
|  | Constant | 0.002*** (0.000) | - | - | - |
|  | Stock return network | 0.477*** (0.003) | 0.000 | 0.000 | 1.000 |
|  | *F-test* | 106,606.260*** | 0.000 | 0.000 | 1.000 |
|  | *R^2^* | 0.264 |  |  |  |

Number of observations $N(N-1)=215,760$; standard error for each coefficient in parentheses; MR QAP – Double-Semi-Partialing (DSP) method; number of iterations for each model: 46,500; *** Denotes significance at the 0.1% level (based on standard regression analysis)

Table 12. Critical threshold for robustness to failure (weighted networks)

| **Network** | SRN | FMN | Layer 1 | Layer 2 | Layer 3 |
| --- | --- | --- | --- | --- | --- |
| $\boldsymbol{f}_{\boldsymbol{c}}$ | 0.724 | 0.852 | 0.339 | 0.684 | 0.724 |

**Comment 7:** *“The differences in statistical properties found between classical MST, FMN layers and FMN itself are generally a direct consequence of the properties of the MST, in particular its acyclicity. The author himself notes this on several occasions. FMN, on the other hand, has a purely endogenous structure. To appreciate this structure as a whole, it would be useful to provide a readable representation (e.g. using a force-directed layout). However, this method only works if the graph has not been binarized first.”*

**Reply:** The FMN network graph was constructed using a force-directed layout. For this purpose, the ForceAtlas2 algorithm was applied. However, since this type of network visualization requires an unweighted network, the visualization has been included in an additional section, “4.6. Robustness Analysis,” where it is based on an unweighted multiplex network (see comment #6).

**page 37, lines 827-829**

**The following text has been added:**

Figure 7 illustrates the visualization of the multiplex graph (FMN) using a force-directed layout, which was generated with the ForceAtlas2 algorithm (node colors correspond to sector affiliation).

**Fig 7. Weighted multiplex network**

**Comment 8:** *“The result of multiplex network regression analysis (Table 6) is largely determined by the way the problem is framed: even in out-of-sample, the FMN is likely to be better explained than the SRN, since the exogenous variables are the layers of the FMN. Similarly, the Sharpe ratio (Table 8) is necessarily better explained by the FMN indicators, which include volatility and the risk-free rate (which participate in its calculation), than by the SRN indicators, which only include returns.”*

**Reply**: The regression analysis is aimed primarily at assessing the dependency structure of each of the three layers, rather than focusing on evaluating them in terms of explaining the multiplex model (FMN) of the network versus the singleplex model (SRN). The significance of the FMN is demonstrated by the findings of the network regression analysis, which show that the different network layers provide unique information to enrich the multiplex dataset. The differences between models 1 and 2 (for FMN and SRN, respectively) indicate that distinct information about the stock market's structure is considered by the two networks.

On the other hand, Section 4.7 (Out-of-sample assessment of the dependency structure representation) examines the effect of selected centrality measures from the two compared networks (FMN and SRN) on the out-of-sample Sharpe ratio. The findings reveal that a complex multiplex network structure achieves a higher impact on future stock performance under the risk–return relationship in the financial market than the monoplex network structure. This finding aligns with the reviewer's hypothesis; however, it is important to note that the observed effect was statistically significant and confirmed. In other words, the potential for redundant, statistically insignificant determinants of performance arising from the risk-return relationship and contributed by each of the three layers of the multiplex network was eliminated.

**Comment 9:** *“The study of robustness to network failures is not convincingly justified. If the graph model is relevant and this robustness is found, then the market is resilient to failures (node deletions). But the robustness to failures result alone does not prove the superiority of the model with which it is obtained, contrary to what is claimed.”*

**Reply:** Appropriate modifications have been implemented in the abstract, Section 4.5, and the conclusions.

**Abstract, lines 11-12**

“Furthermore, the financial multiplex network outperforms the singleplex network of stock returns in terms of robustness to the network falling apart after the random node removal, and it has a structure that better determines the future Sharpe ratio.”

**are revised into,**

“Furthermore, the financial multiplex network outperforms the singleplex network of stock returns ~~in terms of robustness to the network falling apart after the random node removal, and~~ because it has a structure that better determines the future Sharpe ratio.”

**page 36, lines 813-819**

“Table 9 reveals that the FMN has more robust structure to random failures. In order for the financial multiplex network to fall apart, one would have to remove 84% of its vertices, while to damage the giant component of the stock return network, it is enough to randomly remove 58.6% of the nodes. It should be emphasized that the result robustness of the SRN is consistent with those obtained by [95], where for the MST-based cross-correlation network the critical robustness coefficient is $f_{c}=0.61$ and $f_{c}=0.56$, respectively, depending on the period.”

**are revised into,**

“Table 9 reveals that the FMN exhibits ~~has more~~ the most robust structure against random failures. In order for the financial multiplex network to fall apart, one would have to remove 84% of its vertices, while to damage the giant component of the stock return network, it is enough to randomly remove 58.6% of the nodes. It should be emphasized that the result robustness of the SRN is consistent with those obtained by [95], where for the MST-based cross-correlation network the critical robustness coefficient is $f_{c}=0.61$ and $f_{c}=0.56$, respectively, depending on the period. This suggests that the financial market demonstrates resilience to failures in the aftermath of random stock removal.”

**pages 42-43, lines 937-949**

“The out-of-sample evaluation of the dependency structure representation indicates that the financial multiplex network has a greater impact than the stock return network on the future stock performance under the return-risk ratio in the financial market. This statement provides a clear answer to the third research question. Nevertheless, the low values of the determination coefficient ($adjusted R^{2}<5\%$) of the proposed regression models indicate the necessity for further research in this area. It should be pointed out that the observed transitivity, higher density and much smaller fraction of pendant nodes in the FMN compared to the SRN indicate that the proposed multiplex representation has less network fragility to random removal of vertices or edges. This ultimately leads to the fragmentation of the network into two or more distinct components. Moreover, FMN is more robust to the collapse of the largest component due to the removal of randomly selected nodes. The aforementioned conclusions provide a clear answer to the third research question.”

**are revised into,**

““The out-of-sample evaluation of the dependency structure representation indicates that the financial multiplex network has a greater impact than the stock return network on the future stock performance under the return-risk ratio in the financial market. This statement provides a clear answer to the third research question. Nevertheless, the low values of the determination coefficient ($adjusted R^{2}<5\%$) of the proposed regression models indicate the necessity for further research in this area. ~~It should be pointed out that the observed transitivity, higher density and much smaller fraction of pendant nodes in the FMN compared to the SRN indicate that the proposed multiplex representation has less network fragility to random removal of vertices or edges. This ultimately leads to the fragmentation of the network into two or more distinct components. Moreover, FMN is more robust to the collapse of the largest component due to the removal of randomly selected nodes.~~ ~~The aforementioned conclusions provide a clear answer to the third research question.~~”

**Minor remarks:**

**Comment 1:** *“*Figures are difficult to read (non-vector format, low resolution, no sector identification by node color, no asset tickers even for hubs).*”*

**Reply:** All figures have been corrected for resolution using the PACE correction tool, which is provided by PLOS to help prepare figures for submission and ensure high publication-quality standards. However, in the constructed PDF file of the revised manuscript, the drawings are still of reduced quality. Only after clicking the link in the upper right corner does the original drawing open in high quality.

The issue of graphically representing the connections of stocks with respect to sector affiliation has been addressed.

**Figs 3 and 4 have been changed:**

**Fig 3. The MST networks of a) idiosyncratic return; b) volatility; c) trading volume; and d) multiplex network**

**Fig 4. The MST network of stock returns (monoplex network)**

Another issue is the input of node labels with the corresponding ticker. This is particularly challenging with respect to Fig. 3, as it presents four networks on a single figure: the FMN and the three layers from which the multiplex network was constructed. Furthermore, each of these networks contains 465 nodes, which significantly complicates the possibility of adding such additional labels. It should be emphasized that the purpose of Fig. 3 is to present the general structure of these networks. Therefore, the identification of hub nodes in all networks was addressed in the following manner (last row of Table 1). I hope that such a solution is acceptable.

**page 22, Table 1**

**Table 1. Network indicators**

| Network  Indicator | Stock return | Layers | | | Multiplex |
| --- | --- | --- | --- | --- | --- |
|  |  | **Idiosyncratic return** | **Volatility** | **Trading volume** |  |
| Number of nodes $\left( \boldsymbol{N} \right)$ | 465 | 465 | 465 | 465 | 465 |
| Number of edges $\left( \boldsymbol{m} \right)$ | 464 | 464 | 464 | 464 | 1 056 |
| Degree $\left( \boldsymbol{k} \right)$ | 928 | 928 | 928 | 928 | 2 112 |
| Mean degree $\left\langle\boldsymbol{k} \right\rangle$ | 1.996 | 1.996 | 1.996 | 1.996 | 4.542 |
| Maximum degree $\left( \boldsymbol{k}_{\boldsymbol{max}} \right)$ | 13 | 10 | 13 | 26 | 35 |
| Fraction of pendant nodes $\left( \boldsymbol{FPN} \right)$  (number of nodes with degree $\boldsymbol{k=1}$ in parentheses) | 0.538  (250) | 0.454  (211) | 0.574  (267) | 0.626  (291) | 0.028  (13) |
| Network density $\left( \boldsymbol{\rho} \right)$ | 0.004 | 0.004 | 0.004 | 0.004 | 0.010 |
| Mean geodesic distance $\left( \boldsymbol{L} \right)$ | 15.048 | 19.698 | 12.703 | 10.384 | 4.201 |
| Network diameter $\left( \boldsymbol{s} \right)$ | 41 | 46 | 33 | 25 | 9 |
| Graph radius $\left( \boldsymbol{r} \right)$ | 21 | 23 | 17 | 13 | 6 |
| Clustering coefficient $\left( \boldsymbol{C} \right)$ | 0.000 | 0.000 | 0.000 | 0.000 | 0.152 |
| Transitivity $\left( \boldsymbol{T} \right)$ | 0.000 | 0.000 | 0.000 | 0.000 | 0.076 |
| Network tree length $\left( \boldsymbol{NTL} \right)$ | 0.727 | 0.939 | 0.708 | 0.940 | - |
| Mean occupation layer $\left( \boldsymbol{MOL} \right)$ | 9.622 | 13.854 | 8.559 | 7.888 | 2.834 |
| Network degree centralization index $\left( \boldsymbol{NDCI} \right)$ | 2.382% | 1.733% | 2.382% | 5.196% | 6.593% |
| Network closeness centralization index $\left( \boldsymbol{NCCI} \right)$ | 7.552% | 5.855% | 9.559% | 11.712% | 22.186% |
| Network betweenness centralization index $\left( \boldsymbol{NBCI} \right)$ | 62.474% | 63.314% | 63.749% | 70.265% | 18.493% |
| Modularity $\left( \boldsymbol{Q} \right)$ | 0.904 | 0.902 | 0.901 | 0.902 | 0.555 |
| Number of communities $\left( \boldsymbol{NC} \right)$ | 19 | 19 | 20 | 20 | 14 |
| Exponent of power-law $\left( \boldsymbol{\gamma} \right)$  ($\boldsymbol{p}$-value in parentheses) | 3.023  ($p$=0.520) | 4.921  ($p$=0.691) | 2.534  ($p$=0.836) | 2.781  ($p$=0.117) | 3.029  ($p$=0.714) |
| Hub node tickers, based on 99th percentile (degree in the parentheses) | APH (13); MSFT (12); AME (11);  AMP (11);  PRU (10) | PRU (10);  CMS (9);  SFG (8);  ITW (7);  PEP (7) | AME (13); ETN (13); HON (12); ADBE (11); DRI (11) | AMP (26); MSFT (26); PRU (21); XOM (14); WEC (12) | AMP (35); MSFT (30); PRU (29); ETN (21); AME (19) |

**Comment 2:** *“The definition of the content of the multiplex layers comes late (third page of the introduction).”*

**Reply:** The definition of the content of multiplex layers has been moved to the second paragraph of the Introduction.

**pages 2-3, lines 46-50**

“(…). Therefore, it is crucial to analyze financial markets through the lens of multilayer networks rather than isolated monoplex networks, which identifies the research gap. This study provides the backbone for a novel approach, and assumes that the atomistic view of relationships in the financial market is no longer valid.”

**are revised into,**

“(…). Therefore, it is crucial to analyze financial markets through the lens of multilayer networks rather than isolated monoplex networks, which identifies the research gap. This study provides the backbone for a novel approach, and assumes that the atomistic view of relationships in the financial market is no longer valid. For the purposes of this study, the multiplex network structure includes layers representing different time series-based asset relationships, namely, the firm-specific factor of the log-stock return, the volatility of the stock return, and the stock market turnover.”

**page 3, lines 63-70**

“(…). Unlike traditional network models that represent the system as a single layer of nodes and edges, a multilayer network captures multiple interconnected layers, each representing a different aspect of the system. In other words, monoplex network theory is not sufficient to model and explain the complex multi-faceted relationships of the financial market. For the purposes of this study, the multiplex network structure includes layers representing different time series-based asset relationships, namely, the firm-specific factor of the log-stock return, the volatility of the stock return, and the stock market turnover.”

**are revised into,**

“(…). Unlike traditional network models that represent the system as a single layer of nodes and edges, a multilayer network captures multiple interconnected layers, each representing a different aspect of the system. In other words, monoplex network theory is not sufficient to model and explain the complex multi-faceted relationships of the financial market. ~~For the purposes of this study, the multiplex network structure includes layers representing different time series-based asset relationships, namely, the firm-specific factor of the log-stock return, the volatility of the stock return, and the stock market turnover.~~”

**Comment 3:** *“It is understandable that the returns layer should be built from returns purged of the common component, to avoid too strong a correlation with the volatility layer. However, the approach of searching for the common component of the specific factors of returns is partly counter-intuitive and deserves to be discussed. Furthermore, the low similarity of the volatility and volume layers raises questions about indicators of both variables. It is likely that the similarity of the distribution tails would be much stronger. This observation brings us back to the absence of any prior study of the non-linearities of the data used. The proposed literature review also mentions various works focusing on distribution tails and extreme risks.”*

**Reply:** The justification for applying the idiosyncratic stock return layer to a multiplex network has been extended.

**pages 5-6, lines 98-126**

“(…). Idiosyncratic return analysis plays a crucial role in understanding the unique characteristics and performance of individual assets, as demonstrated in the following studies [23–26]. Therefore, the idiosyncratic return network offers a superior representation compared to the conventional network, which relies on total stock return, as evidenced in the following studies [27–30]. In addition, a network based on total return, compared to the idiosyncratic return network, replicates to a greater extent the information received from the volatility network. The exclusion of systematic risk for the first layer of the multiplex network is intended to diversify the three-layer network, in order to avoid the redundancy of information carried by the second layer (volatility network).”

**are revised into,**

“(…). Idiosyncratic return analysis plays a crucial role in understanding the unique characteristics and performance of individual assets, as demonstrated in the following studies [40–43]. El-Nader and Al-Halabi [42] observe a positive idiosyncratic premium return for the UK financial market. The advantage of analyzing idiosyncratic return networks over the stock return network lies in its ability to capture individual asset-specific characteristics and interactions. This granularity allows for a more nuanced understanding of the relationships between individual assets and their contributions to overall market behavior. Therefore, the idiosyncratic return network offers a superior representation compared to the conventional network, which relies on total stock return, as evidenced in the following studies [44–47]. The idiosyncratic return network offers a superior representation compared to conventional networks by providing a more refined and accurate depiction of the relationships between financial assets. This advantage arises from the idiosyncratic return network's ability to isolate the unique risk and return characteristics of each asset, effectively filtering out the influence of systematic factors such as market-wide or sectoral trends. By focusing on residual returns, the idiosyncratic return network eliminates systematic bias, reducing noise and enhancing the detection of meaningful, asset-specific connections. This results in a clearer and more precise understanding of the intrinsic dependencies between assets. Conventional networks, which incorporate both systematic and idiosyncratic risks, often overestimate correlations driven by external factors, thereby obscuring genuine interdependencies. By concentrating on asset-specific characteristics and avoiding distortions introduced by common systematic factors, the idiosyncratic return network enhances the practical applicability of network-based insights in financial decision-making. Therefore, the idiosyncratic return network represents a significant advancement in the study and application of financial network analysis, offering deeper insights into asset interdependencies. In addition, a network based on total return, compared to the idiosyncratic return network, replicates to a greater extent the information received from the volatility network. The exclusion of systematic risk for the first layer of the multiplex network is intended to diversify the three-layer network, in order to avoid the redundancy of information carried by the second layer (volatility network).”

-------------------------------------------------------------------------------------------------------------------------

In the context of the low similarity between the volatility and volume layers, it should be noted that the low similarity between the volatility and volume layers in a multiplex network can be considered a positive phenomenon because it reflects the distinct and complementary information that these layers contribute to the overall understanding of the financial system. If the layers were highly similar, the multiplex framework would provide limited additional insights compared to analyzing a single layer.

**page 41-42, lines 922-924**

“(…). In other words, the idiosyncratic stock return network layer determines the stock return network to a greater extent than the other two layers of the multiplex network.”

**are revised into,**

“(…). In other words, the idiosyncratic stock return network layer determines the stock return network to a greater extent than the other two layers of the multiplex network. In general, the low similarity reduces the risk of overfitting and redundancy in multiplex network models. The divergence ensures that each layer contributes distinct structural and relational information, thereby enhancing the robustness and interpretability of the network. This divergence highlights the multiplex network's ability to capture the multifaceted nature of financial markets by incorporating non-redundant perspectives.”

-------------------------------------------------------------------------------------------------------------------------

In the context of financial networks, the tails of distributions refer to extreme values that can represent rare but significant events, such as abrupt price changes (high volatility) or sharp increases in trading volume. If the distribution tails for volatility and turnover volume exhibit substantial similarity, it may indicate that both indicators respond in a similar manner during extreme events. For instance, during market turbulence, high price volatility often coincides with large trading volumes, driven by investor panic, mass sell orders, or abrupt price corrections. However, similarity in the tails does not necessarily translate to similarity in network structures across the entire data range. The fundamental dynamics of volatility and turnover volume in "normal" market conditions may differ significantly, leading to low similarity between the layers. Extreme values, while important, may be considered as specific cases that do not dominate the overall network structure. Thus, greater similarity in the distribution tails may affect local similarity between the layers in the context of extreme events but does not necessarily alter the general observation of low similarity between these layers under normal conditions.

In response to this comment, I have addressed this issue in the limitations of the study.

**page 43, line 950**

“This research has several limitations. (…).”

**is revised into,**

“This research has several limitations. (…). Secondly, the analysis may underestimate the impact of extreme events due to the overall low similarity between the volatility and turnover volume layers. This limitation arises from focusing on the entire distribution rather than explicitly isolating tail dependencies.”

**Comment 4:** *“The notion of noise (in particular p. 4) to characterize graph density is confusing. In the field of dynamic graphs (and signal processing), noise characterizes the volatility of measured data (edges of the computed graph) compared with the true structure of the data (edges of the underlying graph).”*

**Reply:** Thank you for raising attention to this aspect. The sentence concerning the high-density network and its relation to noise was awkwardly phrased. It has been revised in the updated manuscript.

**page 7, lines 147-153**

“By computing the correlation coefficient for all pairs of assets, one can obtain a fully connected network in which all the edges between each pair of stocks are presented. Such high-density networks are characterized by a huge amount of noise. Therefore, it is essential to filter out the relevant information contained in the complex structure of the cross-correlation matrix among the stocks on the financial market.”

**are revised into,**

“By computing the correlation coefficient for all pairs of assets, one can obtain a fully connected network in which all the edges between each pair of stocks are presented. ~~Such high-density networks are characterized by a huge amount of noise.~~ Such fully connected networks inherently contain a significant amount of noise. Therefore, it is essential to filter out the relevant information contained in the complex structure of the cross-correlation matrix among the stocks on the financial market.”

**Comment 5:** *“*The literature review is largely presented as a succession of summaries. It would be more informative to shorten it by grouping the works into major families of methods.*”*

**Reply:** The literature review has been condensed and reorganized.

**pages 10-12, lines 237-276**

“The results obtained using the multilayer network approach reported by [39] suggest positive relationships between macroeconomic networks based on trade and foreign direct investment and the log-return financial market indices network. Zhao et al. [40], using the temporal network framework as a special case of a multilayered network, have built diversified high return and low risk portfolios. Garas and Argyrakis [41] extract a subgraph with the strongest links for the 100 companies with the highest capitalization on the NYSE. Li and Wen [42] investigate the structure of the multiplex network of the guarantee market in China through the lens of the prevention of risk contagion. They have found that the three singleplex networks have a disassortative nature and follow the power-law distribution, but the three different single layers are not similar to each other. The authors of [43] proposed a multi-layered information spillover network among 30 financial institutions, consisting of three layers: return spillover, volatility spillover, and extreme risk spillover. The multilayer network approach is employed in the construction of a risk contagion model for financial institutions [15,44–46].

A recent analysis in [47] proposes a multilayer financial network model by combining three monoplex networks based on i) Spearman’s correlation coefficient, ii) gray relational analysis, and iii) the maximum information coefficient, respectively, for US and Chinese stock markets. Musmeci et al. [48] investigate the multiplex dependency structure of the US stock market using four layers corresponding to the Pearson, Kendall, tail, and partial correlations among assets. Their findings reveal that certain characteristics are specific to the multiplex structure and would remain undetectable if only a separate analysis of particular monoplex networks were to be performed. A broader overview of the multi-relational approach for the financial market is presented in [49]. This study constructs the multilayer network on the basis of four network layers identifying financial and corporate connections: 1) ownership; 2) interlocking directorates; 3) R&D partnerships; and 4) cross-correlation of stock returns. The authors show that there is a non-trivial overlap between these different singleplex networks. Lacasa et al. [50] propose a horizontal visibility graph algorithm to convert a multidimensional time series into the multilayer network. Based on the time series of the stock prices of the 35 largest US companies, they distinguish periods of crisis from periods of financial stability.”

**are revised into,**

“The multilayer network approach is a widely used methodology in financial studies, wherein the constituent layers encompass trade, foreign direct investment, and financial market indices [59]; information spillovers, including return spillover, volatility spillover, and extreme risk spillover [60]; Spearman’s correlation coefficient, gray relational analysis, and the maximum information coefficient [61]; Pearson, Kendall, tail, and partial correlations [54]; as well as ownership, interlocking directorates, R&D partnerships, and cross-correlation of stock returns [62]. The multilayer network approach has also been applied in the construction of a risk contagion model for financial institutions [18,63–65]. Furthermore, Zhao et al. [66], using the temporal network framework as a special case of a multilayered network, and Lacasa et al. [67] proposed a horizontal visibility graph algorithm to convert a multidimensional time series into the multilayer network.

~~The results obtained using the multilayer network approach reported by [59] suggest positive relationships between macroeconomic networks based on trade and foreign direct investment and the log-return financial market indices network. Zhao et al. [66], using the temporal network framework as a special case of a multilayered network, have built diversified high return and low risk portfolios. Garas and Argyrakis [68] extract a subgraph with the strongest links for the 100 companies with the highest capitalization on the NYSE. Li and Wen [69] investigate the structure of the multiplex network of the guarantee market in China through the lens of the prevention of risk contagion. They have found that the three singleplex networks have a disassortative nature and follow the power-law distribution, but the three different single layers are not similar to each other. The authors of [60] proposed a multi-layered information spillover network among 30 financial institutions, consisting of three layers: return spillover, volatility spillover, and extreme risk spillover. The multilayer network approach is employed in the construction of a risk contagion model for financial institutions [18,63–65].~~

~~A recent analysis in [61] proposes a multilayer financial network model by combining three monoplex networks based on i) Spearman’s correlation coefficient, ii) gray relational analysis, and iii) the maximum information coefficient, respectively, for US and Chinese stock markets. Musmeci et al. [54] investigate the multiplex dependency structure of the US stock market using four layers corresponding to the Pearson, Kendall, tail, and partial correlations among assets. Their findings reveal that certain characteristics are specific to the multiplex structure and would remain undetectable if only a separate analysis of particular monoplex networks were to be performed. A broader overview of the multi-relational approach for the financial market is presented in [62]. This study constructs the multilayer network on the basis of four network layers identifying financial and corporate connections: 1) ownership; 2) interlocking directorates; 3) R&D partnerships; and 4) cross-correlation of stock returns. The authors show that there is a non-trivial overlap between these different singleplex networks. Lacasa et al. [67] propose a horizontal visibility graph algorithm to convert a multidimensional time series into the multilayer network. Based on the time series of the stock prices of the 35 largest US companies, they distinguish periods of crisis from periods of financial stability.~~”

**page 13, lines 304-311**

“The results of another study [55] show that, as stocks become increasingly interconnected, their volatility increasingly retains a memory of their past behavior. Millington and Niranjan [56] demonstrate a positive correlation between the volatility and the largest eigenvalue, and a negative correlation with the number of communities. In another study, [57], it is shown that the higher the market volatility, the denser the MST-based network. Long et al. [58] analyze the volatility transmission mechanisms using a complex network approach and show that the connectivity of the network is more fragile to selective removal than to random attacks.”

**are revised into,**

“~~The results of another study [74] show that, as stocks become increasingly interconnected, their volatility increasingly retains a memory of their past behavior. Millington and Niranjan [75] demonstrate a positive correlation between the volatility and the largest eigenvalue, and a negative correlation with the number of communities. In another study, [76], it is shown that the higher the market volatility, the denser the MST-based network. Long et al. [77] analyze the volatility transmission mechanisms using a complex network approach and show that the connectivity of the network is more fragile to selective removal than to random attacks.~~ The results of other studies demonstrate a positive correlation between volatility and the largest eigenvalue, and a negative correlation with the number of communities [75], that higher market volatility corresponds to the denser MST-based network [76], that the connectivity of the network is more fragile to selective removal than to random attacks [77], and, finally, that as stocks become increasingly interconnected, their volatility tends to retain a memory of their past behavior [74].”

**Comment 6:** *“Different stages of data processing should be discussed. In particular, i) the OLS estimator used for the returns layer is almost certainly biased on the long financial series employed (p. 12). To get an idea of this, it would be useful to provide a time-series representation of the residuals; and ii) the choice of volatility measure is not justified.”*

**Reply:** As recommended, a time-series plot of the residuals has been added (see Fig. 2). In general, the OLS estimator is widely used in the literature to separate firm-specific returns from the common factor component based on the CAPM model. This does not negate the reviewer's valuable observation regarding the limitations of the work. Therefore, in response to this comment, I have addressed this issue in the limitations of the study.

I sincerely hope that the aforementioned arguments and the manner in which this valuable suggestion has been addressed will be favorably received by the Reviewer.

Additionally, the rationale behind the selection of the volatility measure has been provided.

**pages 17-18, lines 399-410**

“*Idiosyncratic return network*. The premium of the firm-specific risk is utilized as a proxy for the idiosyncratic return of the company. To separate the firm-specific return from the common factor property, the observed returns are regressed using the ordinary least squares (OLS) method. To compute the idiosyncratic risk premium, the CAPM model is applied to filter out the common factor of the capital market

$r_{i,t}=\alpha_{i}+\beta_{i}\left( r_{m,t}-r_{f,t} \right)+\varepsilon_{i,t}$	(5)

where $r_{i,t},r_{m,t},r_{f,t}$ represent the return of stock $i$ at time $t$, the market return at time $t$, and the risk-free rate at time $t$; $r_{m,t}-r_{f,t}$ refers to the premium of the systematic risk in the capital market; $\alpha_{i},\beta_{i}$ denote the estimated regression coefficient of firm $i$ (the intercept of the security market line) and the exogenous risk of stock $i$, respectively; and $\varepsilon_{i,t}$ corresponds to the residual which represent the idiosyncratic component of the stock return dependent on firm-specific factors. The above are made for the combined time span of data sets 1 and 2.”

**are revised into,**

“*Idiosyncratic return network*. The premium of the firm-specific risk is utilized as a proxy for the idiosyncratic return of the company. ~~To separate the firm-specific return from the common factor property, the observed returns are regressed using the ordinary least squares (OLS) method.~~ To compute the idiosyncratic risk premium, the CAPM model is applied to filter out the common factor of the capital market

$r_{i,t}=\alpha_{i}+\beta_{i}\left( r_{m,t}-r_{f,t} \right)+\varepsilon_{i,t}$	(5)

where $r_{i,t},r_{m,t},r_{f,t}$ represent the return of stock $i$ at time $t$, the market return at time $t$, and the risk-free rate at time $t$; $r_{m,t}-r_{f,t}$ refers to the premium of the systematic risk in the capital market; $\alpha_{i},\beta_{i}$ denote the estimated regression coefficient of firm $i$ (the intercept of the security market line) and the exogenous risk of stock $i$, respectively; and $\varepsilon_{i,t}$ corresponds to the residual which represent the idiosyncratic component of the stock return dependent on firm-specific factors. The above are made for the combined time span of data sets 1 and 2. To separate the firm-specific return from the common factor property, the observed returns are regressed using the ordinary least squares (OLS) method. This approach has been applied in the work of numerous researchers in the field: [40,44,45,47,94,95].”

**page 21, new lines 475-476 and Fig. 2 have been added**

“Fig. 2 depicts a time-series representation of the residuals ($\varepsilon_{i,t}$) derived from Eq. (5), which represent the firm-specific idiosyncratic component of stock returns.”

**Fig 2. Time-series of idiosyncratic components of stock returns**

**page 43, new lines 950-952 have been added:**

“This research has several limitations. Firstly, the OLS estimator was employed to extract the idiosyncratic stock returns, which may exhibit slight limitations in adequacy when applied to long time series.”

**pages 18-19, lines 425-429**

“*Volatility network*. To produce the volatility network, the approach adopted in [52,58] is used, in which, for the daily stock price data, the volatility $\sigma_{i,t}$ for each stock $i$ and trading day $t$ is calculated by utilizing the proxy

$\sigma_{i,t}=2\cdot\left[ \frac{\max_{} \left\{ P_{i,t} \right\}-\min_{} \left\{ P_{i,t} \right\}}{\max_{} \left\{ P_{i,t} \right\}+\min_{} \left\{ P_{i,t} \right\}} \right]$	(8)

where $\max_{} \left\{ P_{i}\left( t \right) \right\}$ and $\min_{} \left\{ P_{i}\left( t \right) \right\}$ are the highest and lowest price of the trading day.”

**are revised into,**

“*Volatility network*. To produce the volatility network, the approach adopted in [71,77] is used, in which, for the daily stock price data, the volatility $\sigma_{i,t}$ for each stock $i$ and trading day $t$ is calculated by utilizing the proxy

$\sigma_{i,t}=2\cdot\left[ \frac{\max_{} \left\{ P_{i,t} \right\}-\min_{} \left\{ P_{i,t} \right\}}{\max_{} \left\{ P_{i,t} \right\}+\min_{} \left\{ P_{i,t} \right\}} \right]$	(8)

where $\max_{} \left\{ P_{i}\left( t \right) \right\}$ and $\min_{} \left\{ P_{i}\left( t \right) \right\}$ are the highest and lowest price of the trading day.

The selection of the above volatility measure employed in constructing the volatility network is grounded in its ability to accurately capture intraday price movements while remaining computationally efficient. Moreover, the methodology employed is supported by a number of considerations. Firstly, it directly measures the magnitude of price movements within a single trading day, thereby providing a robust proxy for realized volatility. Compared to methodologies based solely on returns or closing prices, this approach incorporates data from the entire trading day, enhancing its sensitivity to transient price dynamics. Secondly, the normalization by the sum of the highest and lowest prices ensures the scale independence of the measure, making it suitable for comparing stocks with varying price levels. This property is critical for constructing volatility networks, where relationships between stocks are assessed based on relative rather than absolute volatility levels. Finally, the simplicity of the formula makes it computationally efficient and more robust against the noise often present in high-frequency trading data. By using only the highest and lowest prices, the approach avoids potential biases introduced by closing prices, which may not fully exhibit intraday variability due to market microstructure effects or end-of-day trading behaviors.”

**Comment 7:** *“The acronyms used in the tables (definitions are given below) do not make for optimal readability.”*

**Reply:** The notations in Table 1 have been modified to enhance its readability.

**page 22, Table 1**

**is revised into,**

| Network  Indicator | Stock return | Layers | | | Multiplex |
| --- | --- | --- | --- | --- | --- |
|  |  | **Idiosyncratic return** | **Volatility** | **Trading volume** |  |
| Number of nodes $\left( \boldsymbol{N} \right)$ | 465 | 465 | 465 | 465 | 465 |
| Number of edges $\left( \boldsymbol{m} \right)$ | 464 | 464 | 464 | 464 | 1 056 |
| Degree $\left( \boldsymbol{k} \right)$ | 928 | 928 | 928 | 928 | 2 112 |
| Mean degree $\left\langle\boldsymbol{k} \right\rangle$ | 1.996 | 1.996 | 1.996 | 1.996 | 4.542 |
| Maximum degree $\left( \boldsymbol{k}_{\boldsymbol{max}} \right)$ | 13 | 10 | 13 | 26 | 35 |
| Fraction of pendant nodes $\left( \boldsymbol{FPN} \right)$  (number of nodes with degree $\boldsymbol{k=1}$ in parentheses) | 0.538  (250) | 0.454  (211) | 0.574  (267) | 0.626  (291) | 0.028  (13) |
| Network density $\left( \boldsymbol{\rho} \right)$ | 0.004 | 0.004 | 0.004 | 0.004 | 0.010 |
| Mean geodesic distance $\left( \boldsymbol{L} \right)$ | 15.048 | 19.698 | 12.703 | 10.384 | 4.201 |
| Network diameter $\left( \boldsymbol{s} \right)$ | 41 | 46 | 33 | 25 | 9 |
| Graph radius $\left( \boldsymbol{r} \right)$ | 21 | 23 | 17 | 13 | 6 |
| Clustering coefficient $\left( \boldsymbol{C} \right)$ | 0.000 | 0.000 | 0.000 | 0.000 | 0.152 |
| Transitivity $\left( \boldsymbol{T} \right)$ | 0.000 | 0.000 | 0.000 | 0.000 | 0.076 |
| Network tree length $\left( \boldsymbol{NTL} \right)$ | 0.727 | 0.939 | 0.708 | 0.940 | - |
| Mean occupation layer $\left( \boldsymbol{MOL} \right)$ | 9.622 | 13.854 | 8.559 | 7.888 | 2.834 |
| Network degree centralization index $\left( \boldsymbol{NDCI} \right)$ | 2.382% | 1.733% | 2.382% | 5.196% | 6.593% |
| Network closeness centralization index $\left( \boldsymbol{NCCI} \right)$ | 7.552% | 5.855% | 9.559% | 11.712% | 22.186% |
| Network betweenness centralization index $\left( \boldsymbol{NBCI} \right)$ | 62.474% | 63.314% | 63.749% | 70.265% | 18.493% |
| Modularity $\left( \boldsymbol{Q} \right)$ | 0.904 | 0.902 | 0.901 | 0.902 | 0.555 |
| Number of communities $\left( \boldsymbol{NC} \right)$ | 19 | 19 | 20 | 20 | 14 |
| Exponent of power-law $\left( \boldsymbol{\gamma} \right)$  ($\boldsymbol{p}$-value in parentheses) | 3.023  ($p$=0.520) | 4.921  ($p$=0.691) | 2.534  ($p$=0.836) | 2.781  ($p$=0.117) | 3.029  ($p$=0.714) |
| Hub node tickers, based on 99th percentile (degree in the parentheses) | APH (13); MSFT (12); AME (11);  AMP (11);  PRU (10) | PRU (10);  CMS (9);  SFG (8);  ITW (7);  PEP (7) | AME (13); ETN (13); HON (12); ADBE (11); DRI (11) | AMP (26); MSFT (26); PRU (21); XOM (14); WEC (12) | AMP (35); MSFT (30); PRU (29); ETN (21); AME (19) |

**Comment 8:** *“*In Tables 4 and 5, is it correct that the similarity measures (correlations and Jaccard index) of the layers with the FMN are all identical, whereas the bilateral similarities between layers are different? It would also be useful to calculate the Jaccard of the three layers without the FMN (and not just the three layers and the FMN).*”*

**Reply:** Yes, the similarity measures (correlations and Jaccard index) of the layers with the FMN are indeed the same. This phenomenon arises from the aggregation of three layers, each exhibiting varying degrees of similarity, into a multiplexed form, where a portion of the edges from two or three layers overlap, and each layer contains the same number of edges, equal to N-1=464, as dictated by the properties of the MST approach.

Additionally, as suggested by the Reviewer, the Jaccard index calculations for the three layers have been performed.

**page 31, lines 694-697**

“(…). After conducting 9,300 QAP iterations, it was determined that all the similarity measures in Table 5 exhibit statistical significance. Specifically, the observed matching coefficients are larger than would be expected by random chance with a probability of 1.0.”

**are revised into,**

“(…). After conducting 9,300 QAP iterations, it was determined that all the similarity measures in Table 6 exhibit statistical significance. Specifically, the observed matching coefficients are larger than would be expected by random chance with a probability of 1.0. It is noteworthy that the similarity measures, including Pearson correlation coefficients, the Jaccard index, and Czekanowski-Sørensen-Dice matching coefficients, for each layer with the FMN, yield identical values. This phenomenon results from aggregating three layers with varying similarities into a multiplex form, where some edges overlap across layers, and each layer contains the same number of edges $\left( N-1=464 \right)$ due to the MST properties.”

**pages 31-32, lines 703-716**

“Computing the Jaccard similarity coefficient for the four networks is recommended, considering all three layers and the multiplex network. The formula in the following can be used to extend the Jaccard coefficient to more than two sets

$J_{\left( G_{1},G_{2},\ldots,G_{s} \right)}=\frac{\left| E_{G_{1}}\wedge E_{G_{2}}\wedge\ldots\wedge E_{G_{s}} \right|}{\left| E_{G_{1}}\vee E_{G_{2}}\vee\ldots\vee E_{G_{s}} \right|}$	(16)

where $E_{G_{1}},E_{G_{2}},\ldots,E_{G_{s}}$ represent the edges of each network $\left( s=4 \right)$.

The Jaccard index for all three layers and the multiplex network is 7.95%, which means that, of the 1,056 edges contained in all four networks together, only 84 overlap (84/1,056=0.0795). Fig. 5 depicts a graphical illustration of the edge overlapping for the multiplex network and its three layers, as well as for the FMN with the SRN.”

**are revised into,**

“Computing the Jaccard similarity coefficient for the four networks is recommended, considering all three layers and the multiplex network. The formula in the following can be used to extend the Jaccard coefficient to more than two sets

$J_{\left( G_{1},G_{2},\ldots,G_{s} \right)}=\frac{\left| E_{G_{1}}\wedge E_{G_{2}}\wedge\ldots\wedge E_{G_{s}} \right|}{\left| E_{G_{1}}\vee E_{G_{2}}\vee\ldots\vee E_{G_{s}} \right|}$	(16)

where $E_{G_{1}},E_{G_{2}},\ldots,E_{G_{s}}$ represent the edges of each network, and $s$ $\left( s=4 \right)$ is the number of sets. Table 7 shows the Jaccard index for all three layers.

Table 7. Jaccard index for two or more sets

| **Networks/layers** | **Multiplex** | **Idiosyncratic return & Volatility** | **Idiosyncratic return & Trading volume** | **Volatility & Trading volume** |
| --- | --- | --- | --- | --- |
| **Three layers** | 0.0795 | 0.1152 | 0.1024 | 0.1031 |

The Jaccard index for all three layers and the multiplex network is 7.95%, which means that, of the 1,056 edges contained in all four networks together, only 84 overlap (84/1,056=0.0795). Fig. 5 depicts a graphical illustration of the edge overlapping for the multiplex network and its three layers, as well as for the FMN with the SRN. The Jaccard index for three layers and for each pair of layers ranges from 10.2% to 11.5%. This indicates a higher proportion of overlapping edges within each pair of layers compared to the multiplexed representation.”

**Comment 9:** *“The introduction provides some answers to the research questions mentioned in the introduction: it would be useful to reiterate these questions.”*

**Reply:** The research questions were repeated with the answers given.

**page 41, lines 905-908**

“(…). The inter-layer mutual information shows that the degree distribution is not strongly correlated between the layers of the financial multiplex network. This provides the basis for an affirmative answer to the first research question.”

**are revised into,**

“(…). The inter-layer mutual information shows that the degree distribution is not strongly correlated between the layers of the financial multiplex network. This provides the basis for an affirmative answer to the first research question~~.~~: “Is there a significant difference between the FMN and the SRN?”

**page 42, lines 933-934**

“(…). The above conclusions legitimize the affirmative answer to the second research question.”

**are revised into,**

“(…). The above conclusions legitimize the affirmative answer to the second research question~~.~~: “Does each layer of the stock network offer unique information within the FMN?”

**page 43, lines 947-948**

“(…). The aforementioned conclusions provide a clear answer to the third research question.”

**are revised into (page 42, lines 938-940)**

“(…). This statement provides a clear answer to the third research question~~.~~: “Which network more accurately reflects the interconnections between financial assets?””
