# Supplementary material for: The dependency structure of the financial multiplex network model: New evidence from the cross-correlation of idiosyncratic returns, volatility, and trading volume
Source: PLoS One. 2025 Apr 18;20(4):e0320799. doi: 10.1371/journal.pone.0320799 (PMC12007721; doi:10.1371/journal.pone.0320799)
Supplement: Attachment — Submitted filename: pone.0320799.docx [file pone.0320799.s005.docx]

February 21, 2025

**Point-by-point response to Reviewer**

**Editor comment:** In your next submission, please address the minor comments provided by the reviewer. The editor will assess the revisions, and since these are relatively small changes, the manuscript is not expected to undergo another round of reviews.

**Responses to Editor**

Dear Editor:

Thank you very much for the referee's report on my manuscript “The dependency structure of the financial multiplex network model”. The article has been revised in accordance with the recommendations set forth by the reviewer.

Please find enclosed a detailed, point-by-point response to the reviewer's comments, with all changes clearly specified. Changes in the text are marked in blue.

I wish to take this opportunity to thank the Reviewer for her/his time, effort and insightful comments that have resulted in an improved manuscript. I would also like to thank you for handling my paper. Hopefully, my revised manuscript can be regarded as meeting the high standards of PlosOne journal.

With kind regards

Corresponding author

**Point-to-point responses to the Reviewer**

**Response to Reviewer #2:**

I would like to thank the referee because he/she provided a considerable number of comments to improve my paper. The newly added and significantly revised portions in the paper are marked in red to make them easy to identify.

**Comment 1:** *“All comments have been adequately addressed and justified in detail in the author's reply.*

*In particular, the contribution is now adequately situated, including in relation to the literature in the field of portfolio optimization, the study of the robustness of the proposed graph model is now clearly separated from the notion of efficiency of the portfolios represented, and the readability of the graphs is greatly improved by the use of node colors corresponding to the industries. This last point also makes it possible to verify that clustering by industry does indeed exist, which is an expected property of financial networks.*

*I simply suggest displaying on the graphs the tickers of assets exceeding a centrality threshold to be defined. For example, in figure 7, the graph has an atypical structure. A node is isolated on the right-hand side of the figure. It is characterized by both a very high degree and a very large distance from other nodes, which constitutes a highly uncommon association. It would be useful to know which asset it is to assess the reasons for this.”*

**Reply:** Thank you for the positive feedback on the addressed revisions and for the additional remark. The network structure in Figure 7 is indeed uncommon. In accordance with the suggestion, ticker labels have been added in Figure 7 for the four assets whose degree centrality (in the weighted network of the multiplex graph) exceeds the threshold (degree centrality > 0.05).

**page 35, lines 777-787**

“Figure 7 illustrates the visualization of the multiplex graph (FMN) using a force-directed layout, which was generated with the ForceAtlas2 algorithm (node colors correspond to sector affiliation). It should be stressed that comparisons of the statistical properties of the networks were omitted, as weighted and unweighted networks inherently differ. The robustness test is applied to the analyses presented in Sections 4.3, 4.4, and 4.5.”

**is revised into,**

“Figure 7 illustrates the visualization of the multiplex graph (FMN) using a force-directed layout, which was generated with the ForceAtlas2 algorithm (node colors correspond to sector affiliation). In the presented graph, four companies (PRU, MSFT, AMP, ETN) have been highlighted, as their degree centrality in the weighted multiplex network exceeds the threshold value of 0.05 (degree centrality > 0.05). Two of these firms belong to the Financial Services sector, while one represents the Technology sector and another the Industrials sector. Notably, the AMP node stands out due to both its high degree of connectivity and its considerable distance from other vertices.

It should be stressed that comparisons of the statistical properties of the networks were omitted, as weighted and unweighted networks inherently differ. The robustness test is applied to the analyses presented in Sections 4.3, 4.4, and 4.5.”

**Fig. 7**

**has been changed**
